# A Star Polyamine-Based Nanocarrier Delivery System for Enhanced Avermectin Contact and Stomach Toxicity against Green Peach Aphids

**DOI:** 10.3390/nano12091445

**Published:** 2022-04-23

**Authors:** Yanxiao Yang, Qinhong Jiang, Min Peng, Ziyi Zhou, Xiangge Du, Meizhen Yin, Jie Shen, Shuo Yan

**Affiliations:** 1 Department of Plant Biosecurity and MOA Key Laboratory of Surveillance and Management for Plant Quarantine Pests, College of Plant Protection, China Agricultural University, Beijing 100193, China; yyxiao001225@163.com (Y.Y.); j.yucheng@outlook.com (Q.J.); draco18390552985@163.com (Z.Z.); duxge@cau.edu.cn (X.D.); 2 State Key Laboratory of Chemical Resource Engineering, Beijing University of Chemical Technology, Beijing 100029, China; 2020400119@buct.edu.cn (M.P.); yinmz@mail.buct.edu.cn (M.Y.); 3 Beijing Laboratory of Biomedical Materials, Beijing University of Chemical Technology, Beijing 100029, China

**Keywords:** nanocarrier, nanopesticide, pesticide nanometerization, pest management, star polyamine

## Abstract

The unscientific application of synthesized/botanical pesticides has not only brought the resistance of plant pathogens and pests, but also led to serious environmental pollution. In recent years, various nano-delivery systems have been used for the development of environmental-friendly pesticides with improved efficacy. Herein, the current study constructed an efficient avermectin B1a (AVM) nano-delivery system based on a star polyamine (SPc) and focused on the characterization and bioactivity of SPc-loaded AVM at various mass ratios. The hydroxyl groups of AVM could assemble with carbonyl groups of SPc through hydrogen bond and van der Waals forces, and the self-assembly of AVM and SPc formed nearly spherical particles of AVM/SPc complex with nanoscale size. The contact angle of SPc-loaded AVM decreased with the increasing mass ratio of SPc, revealing the easier distribution and spreading of the AVM/SPc complex. Furthermore, the stomach and contact toxicity of AVM/SPc complex also increased along with the increasing SPc mass ratio, which could be attributed to the enhanced systemic transportation in plants, enlarged contact area to insect pests and stronger permeability across the insect cuticle. The current study provides an efficient nano-delivery system for increasing stomach and contact toxicity of pesticides with wide applications in the agricultural field.

## 1. Introduction

The utilization of synthesized/botanical pesticides can promote the sustained and stable growth of agricultural production to satisfy the global food demand for growing world population [1,2]. However, the excessive application of pesticides has not only brought the resistance of plant pathogens and pests, but also led to serious environmental pollution such as the loss of biodiversity, eutrophication of water bodies, harmful residues on crops, etc. [3,4,5]. The majority of pesticides are hydrophobic, and thus extensive amounts of organic solvents should be used for solubilization and dispersal during spraying processes [6,7]. Thus, the application of pesticides is a double-edged sword, and future research directions should increase the utilization efficiency of pesticides and reduce their potential negative effects on environmental and human health.

In recent years, various nanoparticles have been widely used for the development of environmentally friendly pesticides with improved efficacy, targeted-delivery, controlled-release and enhanced stability characteristics [8,9,10,11]. One of the key advantages of nanopesticides is their nanoscale particle size. This feature can reduce the self-aggregation of pesticides, which is beneficial for the uptake of active ingredients (AIs) by plant pathogens, insect pests or even plants for enhanced bioactivity [12,13,14]. Our group has constructed a star polyamine (SPc) that can be applied as gene/pesticide nanocarrier to improve their delivery efficiency [15]. The SPc can combine with exogenous agents through various interaction forces and activate the clathrin-mediated endocytosis for enhanced uptake and delivery [16,17]. The application of SPc can increase the plant uptake and control efficacy of several pesticides, and reduce the pesticide residue simultaneously [18,19,20]. Thus, the SPc has the characteristics of environmental friendliness, biodegradability and low production cost, which is a suitable adjuvant/nanocarrier to delivery synthetic/botanical pesticides for improved bioactivity.

Avermectins refer to a new class of bio-insecticide macrocyclic lactones that are derived from the secondary metabolites of *Streptomyces avermitilis* [21]. As a major component of avermectins, avermectin B1a (AVM) is widely applied to control internal anthelmintic, external parasites and agricultural pests, exhibiting broad-spectrum bioactivities. It is well known that AVM mainly disrupts the nervous system potentiating GABA-related chloride ion channels and glutamate-gated chloride channels [22]. AVM has a half-life of only 3 h after ultraviolet irradiation; thus, most studies have focused on the ultraviolet protection and controlled release of AVM [23,24,25,26,27,28,29]. However, AVM nanometerization has not been successfully explored since that AVM self-aggregates to large particles.

In this context, an efficient AVM nano-delivery system was constructed to reduce the particle size and contact angle as well as to enhance plant uptake for improvement of pest stomach and contact toxicity. The current study focused on the characterization and bioactivity of SPc-loaded AVM at various mass ratios. We determined the pesticide loading content of SPc, analyzed the particle size and morphology of AVM/SPc complex at various mass ratios, and tested the interaction between AVM and SPc to elucidate the self-assembly mechanism of AVM/SPc complex. The contact angle of SPc-loaded AVM at various mass ratios was also evaluated, and their stomach and contact toxicity against green peach aphids was also determined through root application and immersion methods, respectively. The outcome of this study provides an efficient nano-approach for improving stomach and contact toxicity of pesticides with wide applications in the agricultural field.

## 2. Materials and Methods

### 2.1. Chemical Reagents

Pure AVM B1a (≥97%) was purchased from Macklin Inc. (Shanghai, China). For SPc synthesis, the N,N,N′,N′,N″-Pentamethyl diethylenetriamine (PMDETA, 98%) and CuBr (99.999%) were purchased from Sigma-Aldrich (Saint Louis, MO, USA), the 2-bromo-2-methylpropionyl bromide and triethylamine were purchased from Heowns BioChem Technologies (Tianjin, China), and the 2-(Dimethyl amino) ethyl methacrylate (DMAEMA, 99%) was purchased from Energy Chemical (Shanghai, China). Other agents such as ethanol, methanol, etc. were purchased from Beijing Chemical Works (Beijing, China).

### 2.2. SPc Construction

**Figure 1:**
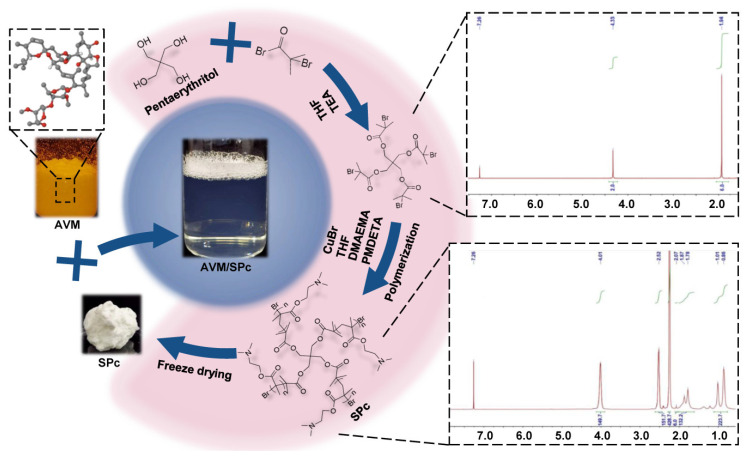
Synthesis route of SPc and preparation of AVM nano-delivery system.

The SPc was synthesized based on the commercial and cheap material sources through two reaction steps according to the method described by Li et al. [15] (Figure 1). For the synthesis of star initiator (Pt-Br), the 2-bromo-2-methylpropionyl bromide (253 mg, 1.11 mmol) was added dropwise into the pentaerythritol solution (25 mg, 0.18 mmol) in dry tetrahydrofuran (THF, 20 mL) and triethylamine (TEA, 111.3 mg, 1.11 mmol) at 0 °C, and the mixture was stirring for 24 h at room temperature. The reaction was quenched with methanol, and the product was recrystallized in cold ether to obtain the Pt-Br (50 mg, 40%), which was confirmed by ^1^H NMR (CDCl_3_, Bruker 400). For the further synthesis of SPc, a flask equipped with a magnetic stirrer was charged with the Pt-Br (40 mg, 0.055 mmol), DMAEMA (2.2 g, 7.7 mmol) and dry THF (8 mL), and the mixture was degassed by nitrogen for 30 min. The CuBr (46 mg, 0.22 mmol) and PMDETA (110 mg, 0.44 mmol) were then added, and the polymerization was carried out in an oil bath at 60 °C for 7 h. The reaction was quenched by cooling and air exposure, and the THF was removed and recycled for the next polymerization. The crude polyamine was purified by dialysis in water, and the white powder of SPc was finally obtained. The structure of SPc was also confirmed by ^1^H NMR (CDCl_3_, Bruker 400).

### 2.3. Preparation of AVM Nano-Delivery System

As shown in Figure 1, 100 mg of pure AVM and SPc were dissolved in 10 mL of ethanol and double distilled water (ddH_2_O), respectively to prepare the mother solutions of AVM and SPc (10 mg/mL). The AVM solution was mixed with SPc solution at mass ratios of 1:1, 1:3 and 1:9, and the mixture was incubated for 15 min at room temperature to prepare the AVM nano-delivery system. The further dilutions were performed using ddH_2_O. The SPc could spontaneously assemble with AVM to form AVM/SPc complex in aqueous solution similarly to other synthetic/botanical pesticides [18,19].

### 2.4. Loading Capacity Measurement

Pure AVM was dissolved in ethanol to prepare a series of AVM dilutions with the concentration of 0, 10, 15, 20, 25 and 30 μg/mL, and the ultraviolet absorbance of each sample was determined via UV-vis spectrophotometry (Thermo Genesys180, Saint Louis, MO, USA). The AVM concentration was proportional to the ultraviolet absorbance at 245 nm; thus, the standard calibration curve was constructed using the absorbance at 245 nm. The 0.2 mL of excess AVM (10 mg/mL) was mixed with 0.5 mL of SPc aqueous solution (10 mg/mL). The mixture incubated for 15 min was diluted with ddH_2_O to the total volume of 10 mL, and then dialyzed using the regenerated cellulose with a molecular weight cutoff of 1000 Da (Shanghai Yuanye Bio-Technology Co., Ltd., Shanghai, China) for 12 h. The freeze-dried mixture was dissolved in 18 mL ethanol, and the absorbance at 245 nm was measured to determine AVM concentration. The pesticide-loading content (PLC) was calculated using the formula of PLC (%) = weight of AVM loaded in complex ÷ weight of AVM-loaded complex × 100%. Each treatment was repeated 3 times. Meanwhile, the AVM was mixed with SPc according to the PCL, and the ultraviolet absorbance of mixture of AVM and SPc was also tested.

### 2.5. Complex Morphology Characterization and Particle Size Measurement

The morphologies of AVM and AVM/SPc complex at the mass ratios of 1:1, 1:3 and 1:9 were examined using a scanning electron microscopy (SEM, JSM-7500F, JEOL, Tokyo, Japan) with an accelerating voltage of 3 kV. Each sample was dropped on the surface of silica, dried naturally, and coated with a thin layer of platinum for 30 s with ETD-800 sputter coater (Beijing Elaborate Technology Development Ltd., Beijing, China). The particle sizes and polydispersity of above samples were also measured using a Particle Sizer and Zeta Potential Analyzer (Brookhaven NanoBrook Omni, New York, NY, USA) at 25 °C. Each treatment contained 3 independent samples.

### 2.6. Isothermal Titration Calorimetry (ITC) Assay

The binding force between AVM and SPc was detected to illustrate the self-assembly mechanism using ITC that is regarded as a high-accuracy method for measuring binding affinities [30,31]. The 2 mL of pure AVM solution (0.138 mmol/L) was titrated with 250 μL of SPc solution (1 mmol/L) in Nano ITC (TA Instruments Waters, New Castle, DE, USA). The heats of interaction during each injection were calculated by integrating each titration peak using Origin7 software (OriginLab Co., Ltd., Northampton, MA, USA). The test temperature was 25 °C, and ΔG was calculated using the formula of ΔG = ΔH − TΔS.

### 2.7. Contact Angle Analysis

The utilization efficiency of pesticides with efficient deposition and strong adhesion to the leaf surface is usually high in the actual production [32]. The contact angles of AVM and AVM/SPc complex at mass ratios of 1:1, 1:3 and 1:9 were examined to evaluate the wetting performance using an Optical Contact Angle Meter (Date Physics Corporation OCA25, Stuttgart, Germany) according to the method described by Zhu et al. [33]. The 5 μL of various formulations (AVM concentration: 1 mg/mL; ethanol/ddH_2_O = 1:9) was dripped onto the glass slide, and the image of the contact angle was collected when the droplet became stable. The ellipse fitting algorithm was used to analyze the contact angle [34]. The algorithm assumes that the water drop profile is part of an ellipse. Each treatment contained 3 independent samples.

### 2.8. Stomach Toxicity Assay through Root Application

Systematic transmission of pesticides is important for controlling aphids that mainly pierce the phloem and indirectly transmit plant virus in many crops [35]. Thus, the stomach toxicity of SPc-loaded AVM was evaluated against green peach aphids using the root application as described by Deng et al. [36] and Zhang et al. [37]. The radish seedlings infested with aphids were maintained at 18 ± 1 °C, 80 ± 10% relative humidity and 14 L: 10 D photoperiod in an incubator. The roots of 9–10 cm height radish seedlings infested with aphids (about 30 aphids per plant) were immersed in the formulations of AVM and AVM/SPc complex at the mass ratios of 1:1, 1:3 and 1:9 (AVM concentration: 20 mg/L). The highest concentration of SPc and ddH_2_O were used as controls. The number of dead aphids was recorded on 1, 2 and 3 d after the treatment, and the corrected mortality (CM) was calculated using the formula of CM (%) = (mortality in treatment − mortality in control) ÷ (1 − mortality in control) × 100%. Each treatment was repeated 5 times.

### 2.9. Contact Toxicity Assay through Immersion Method

The contact toxicity of SPc-loaded AVM was determined against green peach aphids using leaf immersion method as described by Yan et al. [18]. The tobacco leaves infested with green peach aphids (about 30 aphids per treatment) were immersed in AVM solution and AVM/SPc complex solution at the mass ratios of 1:1, 1:3 and 1:9 (AVM concentration: 0.2 mg/L) for 5 s. The highest concentration of SPc and ddH_2_O were also used as controls. The number of dead aphids was recorded, and the corrected mortality was calculated similarly as above. Each treatment was repeated 5 times.

### 2.10. Data Analysis

The statistical analysis was performed using the SPSS 26.0 software (SPSS Inc., New York, NY, USA). The ANOVA with Tukey HSD test was used to analyze the data at the *p* = 0.05 level of significance. The descriptive statistics were shown as the mean value and standard errors of the mean.

## 3. Results and Discussion

### 3.1. Ultraviolet Absorption of AVM/SPc Complex and Loading Capacity

**Figure 2:**
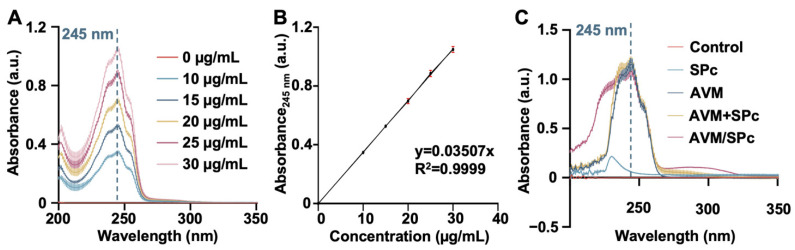
Loading capacity of SPc toward AVM. (**A**) Absorbance spectra of AVM with various concentrations. (**B**) Standard calibration curve of AVM. (**C**) Absorbance spectra of AVM/SPc complex alone AVM, SPc and their mixture.

The UV absorbance at 245 nm is usually used to monitor AVM concentration [38]. In the current study, the calibration curve of AVM was linear in the concentration range of 10–30 μg/mL (Figure 2A). The regression equation was y = 0.03507x, and the correlation coefficient was 0.9999 (Figure 2B). The excess AVM was dialyzed to obtain AVM/SPc complex, and the combination of AVM and SPc did not change the UV-Vis spectra of AVM (Figure 2C). The PLC was calculated to be 9.44 ± 0.47%, which was relatively low compared to those of dinotefuran, osthole and thiamethoxam, revealing the loading ability of SPc toward AVM was not as high as other pesticides [18,19,20]. For pH-responsive release and enhanced UV stability, two composite nanocarriers h-BN and BNNP:PEG/MPTMS have been constructed to deliver AVM with the concentration of loaded AVM of 154.88 and 181.91 mg/g [23].

### 3.2. Characterization of Nanoscale AVM/SPc Complex

**Figure 3:**
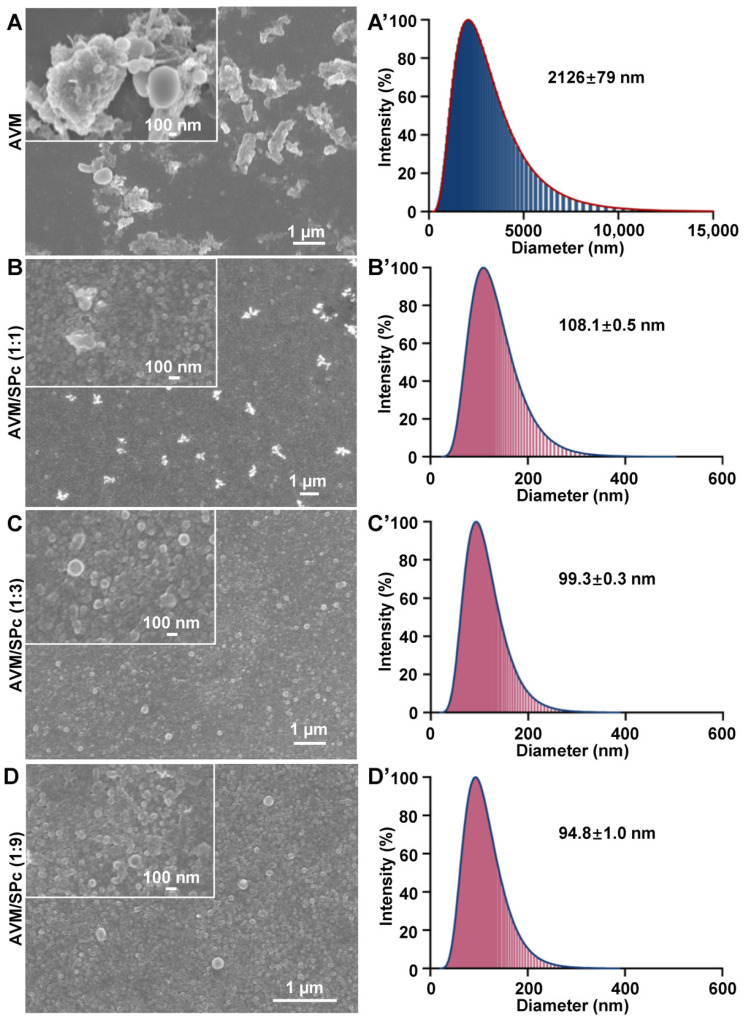
TEM images (**A**–**D**) and particle size distributions (**A’**–**D’**) of AVM (**A**,**A’**)and AVM/SPc complex at the mass ratios of 1:1 (**B**,**B’**), 1:3 (**C**,**C’**) and 1:9 (**D**,**D’**).

Based on the representative SEM images (Figure 3A), AVM self-aggregated into large particles with irregular shape. The particle size of AVM was ranged from 1 to 2 μm, whereas the SEM images of SPc-loaded AVM represented the spherical particles with much smaller size compared to AVM alone. Furthermore, the particle size and characterization of AVM/SPc complex were similar among various mass ratios, and only a small amount of SPc was enough for pesticide nanometerization. This conclusion was also supported by the results of dynamic light scattering (Figure 3B and Table 1). The self-assembly of AVM/SPc complex disturbed the self-aggregated structure of AVM, forming small particles with mean diameter of 108.1 nm (mass ratio of 1:1). There was no significant difference in particle size of AVM/SPc complex among various mass ratios. Meanwhile, the polydispersity of AVM/SPc complex was better than AVM alone. The SPc can be applied as a universal adjuvant for pesticide nanometerization, and the particle sizes of osthole, dinotefuran and thiocyclam can be decreased to 17.66, 29.43 and 52.74 nm with the help of SPc [18,20,39]. The nanometeriztion of SPc-loaded pesticide should not only improve the plant uptake and systemic transmission for enhanced stomach toxicity, but also increase the contact area of pesticide to target pests for enhanced contact toxicity [19,20].

### 3.3. Self-Assembly of AVM/SPc Complex through Hydrogen Bond and van der Waals Forces

**Figure 4:**
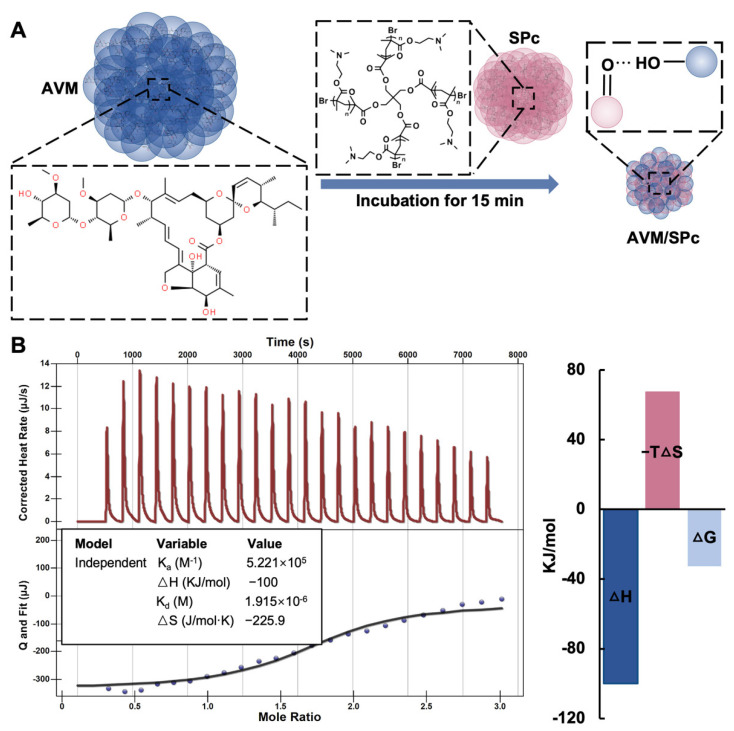
Schematic illustration of the AVM/SPc complex (**A**) and ITC titration of SPc into AVM solution (**B**). The 2 mL of pure AVM solution (0.138 mmol/L) was titrated with 250 μL of SPc solution (1 mmol/L), and the test temperature was 25 °C.

The hydrophobic core of SPc is designed to assemble with hydrophobic AIs, and its hydrophilic shell is beneficial for improving the water solubility and dispersion stability of loaded AIs [15,40]. AVM solution was titrated with SPc solution to determine the binding force to illustrate the self-assembly mechanism (Figure 4). According to the previous interpretation of ITC data [41], a high affinity constant K_a_ of 5.221 × 10^5^ M^−1^ and a low dissociation constant K_d_ of 1.915 × 10^−6^ M suggested that there was an effective and strong interaction between AVM and SPc, and this self-assembly was automatic due to the negative value of ΔG. The negative values of ΔH and ΔS demonstrated that the self-assembly was through hydrogen bond and van der Waals forces. Based on the chemical structures of AVM and SPc, the potential interaction groups might be the hydroxyl groups of AVM and carbonyl groups of SPc. Similar to our previous study, the SPc can conjugate with chitosan and dinotefuran through hydrogen bond and van der Waals forces [16,20]. Meanwhile, the SPc can also assemble with pesticides through other interactions such as hydrophobic association and electrostatic interaction, and the diversity of interactions between SPc and exogenous agents is beneficial for expanding the application area of SPc [19,39,40].

### 3.4. Reduced Contact Angle of AVM/SPc Complex

**Figure 5:**
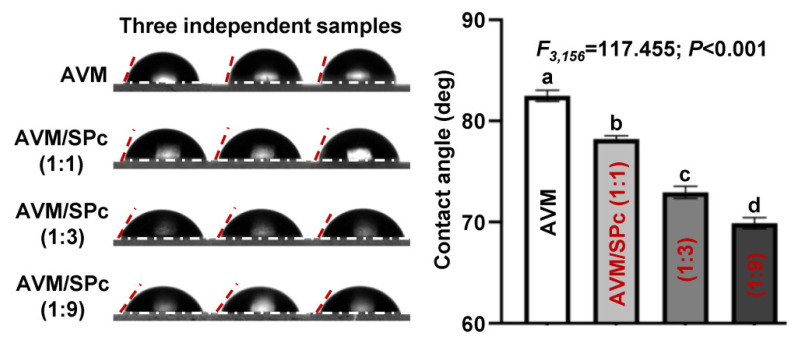
Contact angles of AVM and AVM/SPc complex at the mass ratios of 1:1, 1:3 and 1:9. The 5 μL of various formulations (AVM concentration: 1 mg/mL; ethanol/ddH_2_O = 1:9) was dripped onto the glass slide, and the image of the contact angle was collected. Each treatment contained 3 independent samples. Different letters indicate significant differences (Tukey HSD test, *p* < 0.05).

Both efficient deposition and strong adhesion to the foliage surface are of great importance to minimize loss and raise the utilization efficiency of pesticides, and the contact angle of the pesticide droplet on the foliage surface can reflect its wettability. As shown in Figure 5, after approximately 10 s of the contact, the contact angle of AVM solution was 82.49°, while that of AVM/SPc complex was 78.21° at the mass ratio of 1:1. The contact angle of AVM/SPc complex reduced with the decreasing SPc mass ratio, which could be further reduced to 69.89° at the mass ratio of 1:9. The foliage is mainly composed of trichome and waxy layer to exhibit the hydrophobic characteristic, which leads to the pesticide drift and environmental pollution [42,43]. The potential reason for reduced contact angle may be that (1) the SPc is consisted of the hydrophilic shell that can decrease the surface tension of droplet. (2) The positively charged tertiary amines of SPc are beneficial for the adhesion of loaded pesticides to the leaves that usually carry a net negative charge. Based on the current study, the AVM/SPc complex was more likely to be wetted on plant leaves, and the introduction of SPc could reduce the surface tension of the pesticide droplet to promote its spread and adhesion. Previous studies have designed nanocarriers as pesticide adjuvant for reduced contact angle and surface tension, and enhanced retention [44,45,46,47]. Similar to a previous publication, Chen et al. [32] has modified zein with dialdehyde carboxymethyl cellulose (DCMC) to construct an AVM delivery system, which can regulate the contact angle by adjusting the mass ratio of zein to DCMC. The contact angle of loaded AVM can be decreased from 76° to 63° when the mass ratio of zein to DCMC increases from 2:1 to 4:1. Jia et al. [48] applied adhesive polydopamine microcapsules to load AVM for prolonging foliar pesticide retention, thereby minimizing its volatilization and improving its residence time on crop surfaces.

### 3.5. Improved Stomach Toxicity of SPc-Loaded AVM toward Green Peach Aphids

**Figure 6:**
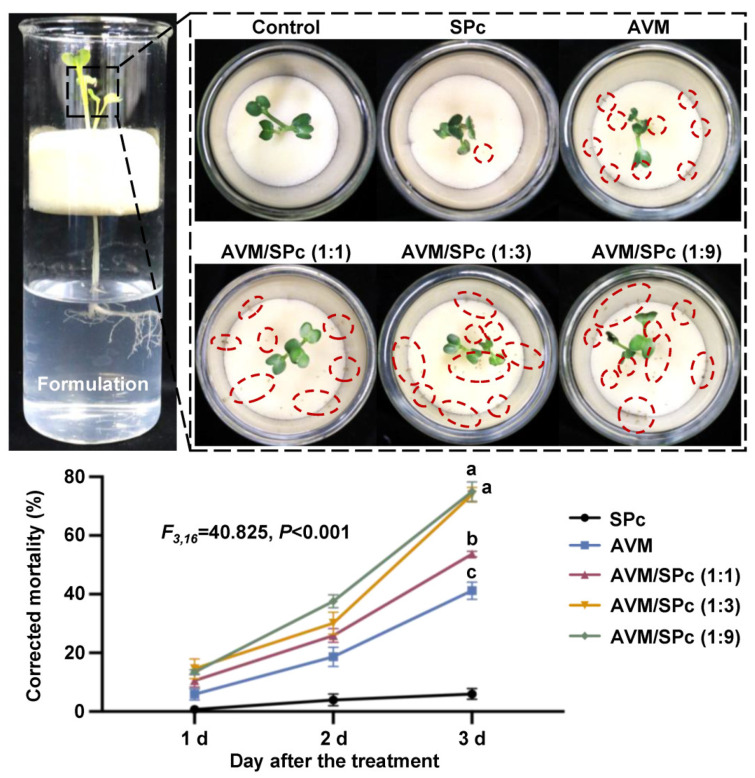
Stomach toxicity of SPc-loaded AVM against green peach aphids through root application. The roots of radish seedlings infested with aphids (about 30 aphids per plant) were immersed in the formulations of AVM and AVM/SPc complex at the mass ratios of 1:1, 1:3 and 1:9 (AVM concentration: 20 mg/L). The highest concentration of SPc and ddH_2_O were used as controls. The red circles indicate the dead aphids. Each treatment was repeated 5 times. Different letters indicate significant differences (Tukey HSD test, *p* < 0.05).

AVM has stomach and contact toxicity against Coleoptera, Lepidoptera and other pests; however, its hydrophobic character constrains the diffusion during the application [49,50,51,52]. Herein, the root application method was firstly used to evaluate the stomach toxicity of SPc-loaded AVM against the green peach aphids (Figure 6). Compared to AVM alone, the stomach toxicity of AVM/SPc complex at various mass ratios increased significantly. More specifically, the corrected mortality of aphids treated with SPc-loaded AVM increased with decreasing mass ratios of AVM to SPc, which was significantly increased by 12.51% (mass ratio of 1:1), 32.71% (mass ratio of 1:3) and 33.75% (mass ratio of 1:9) 3 d after the treatment. The current result was consistent with our previous studies that the mortality of aphids treated with nanoscale thiamethoxam or dinotefuran through the root application was increased by approximately 20% compared with insecticide alone [19,20]. To increase the bioactivity of AVM, Su et al. [52] has constructed an AVM bovine serum albumin nanoparticle, and their stomach and contact toxicity is increased by 35.3% and 19.6%, respectively. The Zein-DCMC-based delivery system can enhance the insecticidal activity of AVM against diamondback moths, with the LC_50_ decreasing from 199.89 to 106.41 mg/L [32].

The systemic transportation of AVM in plants is relatively weak, and only a small amount of AVM can be detected in treated leaves, whereas the AVM can be detected in stems and all leaves of rice plants treated with nanocarrier-loaded AVM, revealing the enhanced transportation [53]. Our results suggested that the SPc could increase the root uptake of AVM and promote AVM to translocate upward in leaves, and the enhanced transportation of AVM by SPc-based delivery system led to the higher bioactivity. The plant uptake of SPc-loaded dinotefuran or thiamethoxam was remarkably improved 1.45–1.53 or 1.69–1.84 times, which might be related to the smaller particle size and reduced contact angle of pesticide/SPc complex [19,20]. Furthermore, the SPc can activate the clathrin-mediated endocytosis by up-regulating *CHMP5*, *Epsin*, *Rab7* gene, etc., to improve the delivery efficiency of loaded cargo [16,17]. Thus, these features of SPc may finally promote the plant uptake-dependent stomach toxicity against aphids.

### 3.6. Improved Contact Toxicity of SPc-Loaded AVM toward Green Peach Aphids

**Figure 7:**
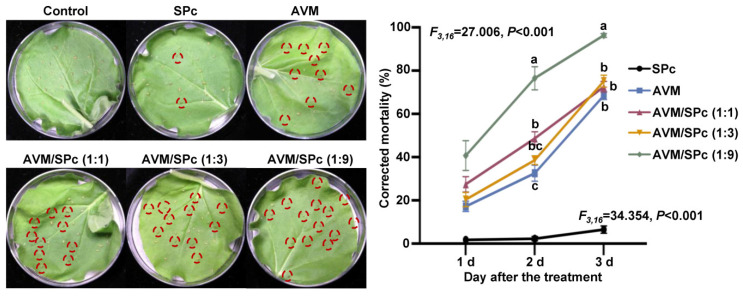
Contact toxicity of SPc-loaded AVM against green peach aphids through immersion method. The tobacco leaves infested with aphids (about 30 aphids per treatment) were immersed in AVM solution and AVM/SPc complex solution at the mass ratios of 1:1, 1:3 and 1:9 (AVM concentration: 0.2 mg/L) for 5 s. The highest concentration of SPc and ddH_2_O were used as controls. The red circles indicate the dead aphids. Each treatment was repeated 5 times. Different letters indicate significant differences (Tukey HSD test, *p* < 0.05).

The immersion method was used to evaluate the contact toxicity of SPc-loaded AVM against the green peach aphids (Figure 7). The dead aphids caused by various formulations exhibited dehydration and turned to black on tobacco leaves. The contact toxicity of AVM/SPc complex at the mass ratio of 1:9 was improved significantly, and the corrected mortality could reach 96.30% compared with 68.50% of AVM alone on 3 d after the treatment. The potential mechanism explaining the enhanced contact toxicity might be due to the efficient nano-delivery system that enlarged the contact area of AVM to aphids. Our previous studies have demonstrated that the SPc-loaded exogenous agents such as double-stranded RNA can penetrate the insect cuticle for efficient delivery [54,55]. This feature of SPc might promote the AVM to penetrate the aphid cuticle for enhanced contact toxicity. A previous study has reported that the emamectin benzoate (EB) nanogel suspension with a polymer poly (vinyl alcohol)-valine exhibits higher anti-pest activity than EB emulsifiable concentrate against diamondback moths, which might be related with the enhanced drug transport across the physiological barriers [56]. As expected, the SPc exhibited no obvious toxicity against aphids, confirming its negligible stomach and contact toxicity. The biotoxicity of SPc has been evaluated using a predatory ladybird, and extremely high concentration of SPc (nearly 9925 times the field application concentration) can down-regulate many membrane protein genes, leading to the damage of cell membrane [57]. Although the SPc exhibits excellent biocompatibility, its potential negative effects on the environment and non-targets should be evaluated in greater detail before the large-scale application in field. Meanwhile, the gene and pesticide co-delivery should be applied to further increase the control efficacy of plant diseases and pests in future [58,59].

## 4. Conclusions

In the current study, a star polyamine was used to successfully construct an efficient AVM nano-delivery system. The AVM could assemble with SPc through hydrogen bond and van der Waals forces, and the potential interaction groups might be the hydroxyl groups of AVM and carbonyl groups of SPc. The interaction between AVM and SPc disturbed the self-aggregated large structure of AVM and thus formed nearly spherical nanoparticles with much smaller particle size. The contact angle of SPc-loaded AVM decreased with the increasing SPc mass ratio, suggesting the easier distribution and spreading of the AVM/SPc complex. Furthermore, the stomach and contact toxicity of AVM/SPc complex increased remarkably compared to AVM alone, and its toxicity increased along with the increasing SPc mass ratio. The potential synergistic mechanism might include the enhanced systemic transportation in plants, enlarged contact area to insect pests and stronger permeability across the insect cuticle, etc. The current study has provided a safe pesticide nano-delivery system for improved stomach and contact toxicity, which is beneficial for pesticide reduction in sustainable agriculture. Extensive studies suggest that the SPc is suitable as a pesticide nanocarrier/adjuvant for green pest management.

## Figures and Tables

**Table 1:** Reduced particle size and polydispersity of SPc-loaded AVM at various mass ratios. Means ± SE followed by different letters are significantly different (Tukey HSD test, *p* < 0.05).

Formulation	Mass Ratio	Sample Number	Polydispersity	Average Polydispersity	Size (nm)	Average Size (nm)
AVM	-	1	0.361	0.428 ± 0.036 ^a^	2077	2126 ± 79 ^a^
		2	0.440		2280	
		3	0.483		2020	
AVM/SPc complex	1:1	1	0.149	0.135 ± 0.009 ^b^	108.6	108.1 ± 0.5 ^b^
		2	0.139		108.6	
		3	0.117		107.1	
	1:3	1	0.091	0.092 ± 0.002 ^b^	99.9	99.3 ± 0.3 ^b^
		2	0.095		99.4	
		3	0.089		98.7	
	1:9	1	0.091	0.082 ± 0.010 ^b^	93.0	94.8 ± 1.0 ^b^
		2	0.063		96.6	
		3	0.093		94.8	
			*F*_3,8_ = 73.659, *p* < 0.001		*F*_3,8_ = 658.503, *p* < 0.001	

## Data Availability

All data in this study will be available from the corresponding author upon reasonable request.
